# Individual abilities to estimate levels of movement synchrony predict action observation network activation

**DOI:** 10.1162/IMAG.a.962

**Published:** 2025-10-24

**Authors:** Ryssa Moffat, Emily S. Cross

**Affiliations:** Social Brain Sciences Lab, ETH Zurich, Zurich, Switzerland; School of Psychological Sciences, Macquarie University, Sydney, Australia; MARCS Institute, Western Sydney University, Sydney, Australia

**Keywords:** embodiment, fNIRS, movement synchrony, action observation network, mirror game

## Abstract

Observers’ ability to estimate levels of movement synchrony, such as in Olympic diving or rowing, is highly variable and, in part, constrained by personality traits and enjoyment of the movements. Embodiment also appears to play a crucial role, whereby stronger beliefs that one’s body can complete physical tasks predict more accurate estimation of synchrony levels. However, to demonstrate the relevance of embodiment per se, direct evidence that physical embodiment (i.e., bodily knowledge of specific movements) alters synchrony estimation is required. Here, we assessed the behavioural and cortical underpinnings of synchrony estimation in conditions with and without physical embodiment. Forty-three participants completed personality trait measures, then completed a synchrony estimation task. Participants copied upper-body movement sequences (acquiring physical embodiment), then viewed dyads performing the same sequence or an unknown sequence and estimated the level of synchrony, while we recorded cortical activation using functional near-infrared spectroscopy (fNIRS). Participants underestimated synchrony levels, showing greater underestimation for unknown movements than performed movements. For performed movements, but not unknown movements, the degree of estimation error was associated with activation of left inferior frontal gyrus and superior temporal gyrus, and right motor cortex and inferior parietal lobule. Participants’ estimation error was associated with body competence and autistic trait scores, as well as measures of enjoyment and movement predictability (replicating previous work). This work demonstrates that physical embodiment improves lay observers’ ability to discern levels of movement synchrony and has practical implications across a wide variety of artistic and athletic domains.

## 1 Introduction

When watching pairs of Olympic divers on television, viewers see the divers count down from three before attempting to launch their bodies into a series of perfectly synchronous rotations before plunging into the water at the exact same moment. While viewers may agree with the judges’ rankings of the first and last place pairs, viewers may find it challenging to appreciate how judges assigned the intermediate rankings. This discrepancy between judges’ and viewers’ certainty about rankings of diver’s synchrony can likely be ascribed to differences in perceptual and embodied experience, gained through extensive observation and physical practice of these movements. We examine the latter in the present study: If a person physically performs a specific movement sequence (i.e., acquiring embodied experience) then watches two other people perform the same sequence, can we expect the person to estimate the level of movement synchrony more accurately than if they had not practiced the sequence? How far is the estimated level of synchrony from the real (objectively measured) level of synchrony?

To date, four studies cumulatively examining more than 700 participants offer robust evidence that lay observers’ abilities to estimate levels of movement synchrony vary substantially (Lumsden et al., 2012; Macpherson et al., 2020; Moffat & Cross, 2024b; Vicary et al., 2017). When an individual estimates levels of movement synchrony, their *accuracy* or, better put, *estimation error* (i.e., difference between estimation and objective measure of synchrony), is statistically linked to their personality traits, self-report measures of embodiment, and enjoyment of the movements (Moffat & Cross, 2024b; Vicary et al., 2017). The relationship between self-report measures of embodiment (described in greater detail in Section 1.4) and estimation error hints at the central role of embodied experience in establishing or strengthening a perceptual sensitivity to movement synchrony. To test the extent to which embodied experience impacts estimation of synchrony empirically, however, we must directly compare participants’ estimation error for movements they have physically performed with their own bodies and movements they have not performed. By contrasting participants’ estimation error in these conditions, the present study moves beyond hinting at the role of embodiment to demonstrate how embodied experience shapes perception of synchrony in multi-person movements.

In the following sections, we briefly describe the literature on movement synchrony and brain networks supporting movement perception, followed by the role of individual differences in embodiment, aesthetic experience, and personality traits in estimating levels of movement synchrony. Finally, we outline how functional near-infrared spectroscopy (fNIRS) can be used to measure patterns of brain activity associated with movement perception and synchrony estimation.

### 1.1 Movement synchrony and its neural underpinnings

Movement synchrony, that is, the alignment of body movements of two or more individuals in space and time, has received substantial empirical attention over the past two decades, with a strong emphasis on the social benefits that synchronous movement, intentional or otherwise, can offer (Koban et al., 2019; Lakin et al., 2003). Falling in step with a peer, clapping in time with a crowd, or matching an interlocuter’s postures during conversation are examples of synchronous movement (Néda et al., 2000; Richardson et al., 2007; Zivotofsky & Hausdorff, 2007) that when temporally aligned are likely to enhance feelings of closeness between people, result in prosocial behaviour, and establish group cohesion in the eyes of ingroup and outgroup members (for meta-analyses, see: Mogan et al., 2017; Rennung & Göritz, 2016; Vicaria & Dickens, 2016).

The great number of social benefits associated with movement synchrony led the phenomenon to be coined “social glue” (Lakin et al., 2003). This colloquial term aptly summarises the central behavioural tenets of Shamay-Tsoory et al.’s (2019) extended integrative model of alignment. In this theoretical model, Shamay-Tsoory and colleagues propose that movement synchrony is one manifestation of the “core mechanism of connectedness.” The authors identify cognitive synchrony and emotional contagion as the other two manifestations. Drawing connections between behaviour and neurophysiology, Shamay-Tsoory and colleagues further describe a network of brain areas involved in the maintenance of these manifestations of connectedness, or interpersonal alignment. The network is composed of three components: a gap-monitoring system, an alignment system, and a reward system. The gap-monitoring system continuously monitors the level of alignment to identify lapses, or gaps, in interpersonal alignment. When a gap in one of the three manifestations of interpersonal alignment is detected by the dorsal anterior cingulate cortex (ACC), dorsal medial prefrontal cortex (PFC), and anterior insula, realignment is initiated and achieved via behaviour matching, which involves the inferior frontal gyrus (IFG), inferior parietal lobule (IPL), premotor cortex (PMC), and superior temporal cortex (STC). In the absence of a gap, the activation of the reward system (ventral striatum, orbitofrontal cortex, and ventral medial PFC) provides the positive feedback needed to sustain alignment. In summary, this theoretical model posits that interpersonal alignment in the form of movement synchrony is socially motivating and self-perpetuating.

The brain areas in Shamay-Tsoory et al.’s (2019) proposed model overlap substantially with the well-established action observation network (AON), which comprises the ventral PMC, IFG, and middle frontal gyrus (MFG), as well as the STC and IPL (Caspers et al., 2010; Cross et al., 2009; Hardwick et al., 2018). The AON supports perception of body movements and initiation of actions, which are both essential functions for maintaining movement synchrony, as demonstrated by neuroimaging studies examining movement imitation (Caspers et al., 2010; Iacoboni, 2005, 2009; Keysers et al., 2018; Mengotti et al., 2012; Molnar-Szakacs et al., 2005). In the context of movement imitation and synchrony, the STC plays two roles: encoding sensory inputs and comparing executed movements to the encoded sensory inputs. Encoded sensory information passes from the STC to the IFG, PMC, and IPL, which in turn encode motor commands and movement goals (Caspers et al., 2010; Iacoboni & Dapretto, 2006; Keysers et al., 2018; Rizzolatti & Sinigaglia, 2016). Cumulatively, these studies demonstrate that the AON is active during performance and observation of movements ranging from simple manual movements to more complex full-body movements, such as dance. Emerging evidence suggests that the AON may also be active during performance and observation of movement performed by one or more people (Cross et al., 2024; Moffat & Cross, 2024a; Orlandi & Candidi, 2025).

### 1.2 The AON, motor simulation, and movement synchrony

Neuroscience research exploring the perception of dance has yielded insights into the extent to which the AON, as measured using fMRI (functional magnetic resonance imaging), encodes expertise and embodied experience with full-body movements (Cross, 2025; Cross & Orlandi, 2020). For example, seminal research by Calvo-Merino et al. (2005) demonstrated greater activation of the AON when expert ballet dancers observe ballet movement, compared with capoeira movements, and vice versa for capoeira performers observing capoeira and ballet movements. Moreover, the AON responds more strongly when expert dancers or non-dancers view full-body movements that they have recently practiced, that is, embodied, as opposed to movements that they have only viewed and not practiced (Cross et al., 2006; Kirsch et al., 2015). One way of interpreting these findings is that the positive relationship between AON activation and the level of the observer’s embodied experience reflects the fidelity of motor simulation, or resonance, processes that occur during movement observation (Cross & Orlandi, 2020; Gallese et al., 2018; Jeannerod, 2001; Keysers et al., 2018). Motor simulation is a critical component in processing, understanding, and even learning the movements one observes (Jeannerod, 2001). Here we use the terms motor simulation and motor resonance to maintain consistency with the brain imaging literature: motor simulation for fMRI and fNIRS studies and motor resonance for EEG and TMS studies.

The most direct form of evidence for motor simulation has been established using neuro-modulatory techniques such as transcranial magnetic stimulation (TMS) to assess motor excitability while observing movements (for review see: Keysers et al., 2018). Typically, researchers apply TMS to the primary motor cortices (MC) or PMC while watching (manual) movement sequences and measure the strength of evoked motor potentials from the contralateral hand muscles (e.g., Fadiga et al., 2005). The amplitude of the measured motor potential is interpreted as an indication of the level of excitability of the motor system (i.e., the strength of simulation or resonance). Another approach is to apply TMS over the AON regions during movement observation and motor planning with the aim to disrupt motor simulation processes performed by these regions. Via such TMS-based approaches, research groups have demonstrated that the strength of motor simulation when watching, or even hearing, another person perform movements is amplified by embodied experience (Hadley et al., 2015) and further shaped by social cues and individuals’ traits (Guidali et al., 2023; Prinsen & Alaerts, 2024).

Hadley et al. (2015) used TMS to examine how motor resonance facilitates turn taking during rehearsed and unrehearsed piano duets. The authors showed that using TMS to disrupt motor processing in the dorsal PMC interfered with observers’ prediction of the timing of rehearsed (i.e., known, embodied) motor sequences performed by another person (Hadley et al., 2015). Guidali et al. (2023) assessed levels of motor resonance while participants observed a hand grasping another hand (i.e., a social object) or a bottle (i.e., a non-social object), and observed selective motor resonance when observing a hand grasping another hand, which was positively associated with participants’ individual empathy scores. Prinsen & Alaerts (2024) reported greater motor resonance when participants viewed an actor performing hand movements facing the participant, as opposed to facing 90˚ away from the participant (i.e., making eye contact vs. looking away)—but only among observers who scored highly on social responsiveness scales. These findings, though diverse, reinforce the role of motor resonance, or simulation, in perceptions of and physical reactions to body movements in social contexts.

### 1.3 Motor simulation of synchronous movements

In the midst of increasing empirical focus on dyadic body movements (for review, see: Orlandi & Candidi, 2025), researchers have compared how AON activity is modulated by movement synchrony vs. asynchrony (Cross et al., 2024; Georgescu et al., 2014). Georgescu et al. (2014) generated videos of simplified human-like avatars performing dyadic movements extracted from real conversations. The 2 x 2 factorial design (synchrony x smoothness) included the avatars performing either asynchronous movements or synchronous movements, and the movements were either smooth or robotic. Participants rated the videos on “naturalness” and were instructed to consider the plausibility and familiarity of such scenes. Georgescu and colleagues found that the duplicated, synchronous movements were rated less natural and evoked less activation in participants’ left IPL, right posterior STS, and right IFG, than asynchronous movements. Based on these findings, the authors suggest that the AON is more sensitive to asynchronous but temporally appropriate movements showing joint agency, as opposed to perfectly synchronous mirrored movements that are less common in social interactions. These instructions to participants (i.e., to consider the plausibility and familiarity of movements) could implicitly frame the synchronous clips as “less natural,” thereby shaping brain responses. Indeed previous research regarding elements of “naturalness” has shown that beliefs about animacy and humanness of agents influence perceptions of movements (Cross et al., 2016; Jastrzab et al., 2024). In the context of this previous work, we draw attention to the fact that using avatars could also impact brain responses to synchrony in a context where perfect synchrony is implicitly framed as unnatural (e.g., Georgescu et al., 2014). Though perfect synchrony, or unison, may be less effective in conversational and collaborative tasks (e.g., Miles et al., 2017; Richardson et al., 2007), perfect synchrony, or unison, can be intentional and desirable in other domains (e.g., Olympic divers, learning sign language in a group class, lifting first aid gurneys).

Extending beyond movement synchrony in conversation, Cross et al. (2024) investigated observers’ perceptions of movement synchrony using videos of two dancers performing synchronous or asynchronous choreographies. Participants rated the movements on how “together” they perceived the dancers to be and how enjoyable the movements were to watch. These instructions could frame synchrony in a positive light. The authors reported that observation of synchronous, relative to asynchronous, movements evoked differential activation in cortical AON regions (greater activation in left IPL and right somatosensory cortex; reduced in right STC and left supplementary motor areas). The authors speculated that these differences may be related to differences in motor simulation for synchronous as opposed to asynchronous movements. Further, Cross and colleagues found that participants’ ratings of the dancers’ togetherness were positively correlated with levels of activation in bilateral dmPFC, IFG, and IPL, as well as left lateral occipital complex and right STS for asynchronous movements. The authors interpreted this as a reflection of upregulated sensorimotor activation plausibly stemming from the motor simulation of multiple people’s non-matching movements.

In light of the instructions to participants, these two studies (Cross et al., 2024; Georgescu et al., 2014) can be interpreted as evidence (i) that cortical AON regions respond differentially to synchrony and asynchrony of body movements performed by dyads and (ii) that AON responses are likely sensitive to appropriateness of the synchrony in the given context. To examine how the brain processes synchronous movements without an influence of a social context, researchers have used electroencephalography (EEG) in conjunction with frequency tagging (e.g., Cracco et al., 2019; Formica et al., 2024). Frequency tagging involves presenting stimuli at specific frequencies and comparing the relative amplitude of the brain’s electrical responses at those frequencies (and their harmonics). Cracco et al. (2022) concatenated still images of a single person performing a series of body postures into dynamic body movements, which they showed in four locations on a computer screen. The four copies of the single person cycled through the dynamic movements at a rate of 1.25 Hz, either synchronously or asynchronously. The findings showed that synchronous movements evoke stronger amplitudes, relative to asynchronous movements, over posterior brain areas. Cracco and colleagues interpreted this to mean that the brain “binds” or “chunks” synchronous body movements together and processes them as a unit, which results in stronger motor resonance.

While the studies described in this section shed light on the brain regions and processes that underpin the processing of dyadic movement belonging squarely to synchronous and asynchronous categories, levels of synchrony in real-world activities are inherently variable. The few studies that do include a variety of levels of synchrony offer robust insights into the interindividual factors that shape perceptions of movement synchrony, as well as individual abilities to estimate levels of movement synchrony (Moffat & Cross, 2024b; Vicary et al., 2017).

### 1.4 Individual differences shape perceptions and estimations of movement synchrony

At the group level, observers’ perceptions of movement synchrony are influenced by kinematic features, such as phase locking and movement fluency (Carlson et al., 2019; Cracco et al., 2022; Hartmann et al., 2019; Miles et al., 2009), as well as subtle visual differences, such as skin tone (Macpherson et al., 2020). At the individual level, observers’ aesthetic perceptions and trait differences are predictive of how well observers estimate synchrony levels (Moffat & Cross, 2024b; Vicary et al., 2017). Measures of *accuracy* or *estimation error* can be calculated by comparing measured and estimated synchrony (Moffat & Cross, 2024b; Vicary et al., 2017).

Vicary et al. (2017) quantified observers’ abilities to explicitly estimate the level of synchrony in multi-person movements and presented evidence that individuals’ abilities vary substantially. The observers viewed live 10-person choreographies and rated the synchrony (called “togetherness”) of the dancers’ movements in real time using a tablet. Observers’ estimations of synchrony levels were then compared with objectively measured levels of synchrony (captured using accelerometers worn on the performers’ wrists), as well as observers’ heart rate and ratings of enjoyment. The analyses comparing perceived and measured movement synchrony revealed that observers’ estimated levels of movement synchrony did not neatly match measured levels of the performers’ synchrony, and in fact, observers tended to underestimate synchrony levels. Vicary and colleagues propose that observers may have rated dancers as being more “together” during periods of the choreography involving greater acceleration and visual change. The objectively measured movement synchrony between performers predicted how much observers enjoyed the performance, as well as changes in observers’ heart rates, reflecting physiological arousal. This research highlighted how individuals can vary in their ability to estimate synchrony levels in observed movements, but did not shed light on the characteristics, traits, or experiences that could explain this variability.

Extending upon Vicary et al.’s (2017) findings, in our previous work, we assessed how individual differences in traits, embodiment, and aesthetic perceptions shape individuals’ abilities to estimate levels of synchrony in dyadic movements (Moffat & Cross, 2024b). More than 300 participants viewed videos of real people playing the mirror game (i.e., matching one another’s spontaneous upper body movement as closely as possible) and completed measures of extraversion, self-esteem, empathy, and autistic traits. We found autistic traits to be most robustly associated with individuals’ degree of estimation error and observed a trending association for self-esteem as well. For the former, an individual’s number of autistic traits as scored by the Comprehensive Autistic Trait Inventory (CATI; English et al., 2021) predicted how strongly an individual underestimated synchrony levels. This finding aligned with existing evidence that reduced sensitivity to biological motion is a feature of autism spectrum disorders (Federici et al., 2020; Todorova et al., 2019). Moreover, this finding does not preclude that participants with more numerous autistic traits may have fixated more strongly, relative to participants with fewer autistic traits, on visually dynamic features (e.g., Irwin & Brancazio, 2014; Klin et al., 2002; Williams & Cross, 2018), such as the moving dyads’ extremities.

With respect to individual differences in embodiment, our previous study also recorded body perception (i.e., self-report measure of the attention one pays to internal bodily functioning), body competence (i.e., self- report measure of one’s belief that one’s body can perform physical tasks), and ratings of how strongly participants believed they could reproduce the observed dyadic movement sequences (Moffat & Cross, 2024b). We found that individuals with greater body competence scores were less likely to underestimate synchrony levels for low-synchrony movements and that participants who rated movements as more reproducible estimated levels of synchrony with less error. Based on these novel findings, we proposed that individuals’ embodied movement experiences shape individuals’ abilities to estimate synchrony levels. However, the self-report and rating-based nature of the employed embodiment measures may limit the generalisability of these findings. To ascertain the degree to which this proposition holds, an empirical approach contrasting embodied experience vs. no embodied experience with specific movement sequences on estimation of synchrony levels is necessary. Moreover, this empirical approach is well suited to offer insights into the role of AON in processing synchronous movements.

### 1.5 Recording neural activation during movement performance and perception

Traditional brain imaging studies have employed fMRI to contrast brain activation when viewing full-body movement sequences associated with embodied experience vs. no embodied experience. However, fMRI is not well suited for measuring brain activation while participants physically practice full-body movements, due to requirements for participants to lie supine with no head movement. By contrast, advances in optical brain imaging have made it possible to record brain activation while participants walk, dance, play drums, and improvise rock music (e.g., Cockx et al., 2024; T. Liu et al., 2021; Ono et al., 2014; Pinti et al., 2020; Tachibana et al., 2019). These studies used functional near-infrared spectroscopy (fNIRS), a non-invasive mobile brain imaging technique that records changes in levels of cortical oxygenation using near-infrared light. Compared with other brain imaging techniques, fNIRS is relatively robust to motion artefacts. This core feature stems from fNIRS’ portability (e.g., large models on rolling trolleys and smaller models in backpacks, on armbands or headbands) relative to immoveable fMRI facilities (Menant et al., 2020). Optimally for the fields of psychology, cognition, and sport science, fNIRS enables researchers to study the cortical correlates of cognition in real-world environments and during real activities, many of which involve movement. We note that mobile EEG offers similar advantages of portability and can provide complementary insights to fNIRS regarding the temporal profile of neural responses while participants move (Barnstaple et al., 2021; Bigand et al., 2025; Rai et al., 2025; Theofanopoulou et al., 2024).

Using fNIRS, in another of our previous studies, we compared cortical activation of AON brain regions while participants mirrored another person’s upper body movements and while they viewed videos of pairs of people playing the mirror game (Moffat & Cross, 2024a). The findings show that activation was greater across all recorded AON regions during performance of movement as compared with observation of movements, and no difference in activation of AON regions during observation of movements that participants had performed once and unknown movement sequences that participants had never seen before. Following from this, we proposed that this unanticipated similarity may be explained by the fact that participants were overarchingly unable to distinguish movement sequences they had performed from unknown sequences and participants believed that they performed most of the sequences.

In this previous work (Moffat & Cross, 2024a), we further focused on the influence of embodied experience (i.e., whether participants had physically performed the movements or not) on cortical responses when viewing synchronous movements, as well as the aesthetic experience of viewing said movements. In terms of cortical activation, performed and unknown movements alike evoked increased cortical activation in right IFG and bilateral STG. Performed and unknown movements also evoked negative BOLD responses in bilateral MFG, PMC, MC, and left IPL, as well as left MC for unknown movements. The degree of cortical activation in left STG was positively associated with participants’ enjoyment of both performed and unknown movements. Ultimately, we found that the observed cortical responses were not sensitive to the embodiment per se but were sensitive to participants’ awareness of having embodied the sequences. In this study, we did not explore the influence of individual differences in participants’ abilities to estimate levels of movement synchrony, despite strong evidence that this ability varies across individuals (Moffat & Cross, 2024b; Vicary et al., 2017).

### 1.6 Current study

In the present study, we undertake a secondary analysis of existing data (Moffat & Cross, 2024a), in which we recorded and compared cortical activation of AON regions while participants mirrored another person’s upper body movements and while participants viewed videos of pairs of people playing the mirror game.

Our first aim is to examine how embodying movements influences the ability to estimate the level of synchrony in observed movements, which we quantify in terms of estimation error. Second, we aim to shed light on the extent to which individual differences in sensitivity to levels of synchrony impact AON responses while participants observe synchronous movements. Third, we seek to replicate our previous findings (Moffat & Cross, 2024b) regarding associations between individuals’ estimation error and interindividual differences in traits, ratings of enjoyment, and recognition of the movements.

## 2 Methods

The data presented here were collected for a previous preregistered study (Moffat & Cross, 2024a). In the preregistration published prior to data collection (https://osf.io/2jn73), we explicitly reserved the option to explore the difference between measured and perceived synchrony (i.e., estimation error) in conjunction with the cortical activation evoked by watching synchronous movements. That is the aim of the present study.

### 2.1 Participants

The dataset includes 45 participants (aged 18–40 years) recruited from the Macquarie University student pool, all of whom provided written informed consent for being included in the study. The inclusion criteria were no history of head injury, no neurological or psychiatric diagnoses, and no current use of psycho-pharmaceutical medication (e.g., Ritalin or SSRIs). Additionally, participants who reported no alcohol consumption within the 12 h prior or tetrahydrocannabinol (THC) use/exposure within the 24 h prior to the experiment were included, in accordance with König et al.’s (2021) protocol. The final sample included 43 participants (mean age = 23.40 ± 5.53 years, 21 female, 21 male, 1 preferred not to say), as two participants’ experimental sessions were unexpectedly interrupted, and the participants were excluded.

Ethical approval for this protocol was obtained from the Macquarie University Human Research Ethics Committee (Ref: 520231287146898). All procedures were completed in accordance with this ethical approval. Participants received course credit or a cash honorarium (AUD $30).

### 2.2 Stimuli

**Fig. 1.**
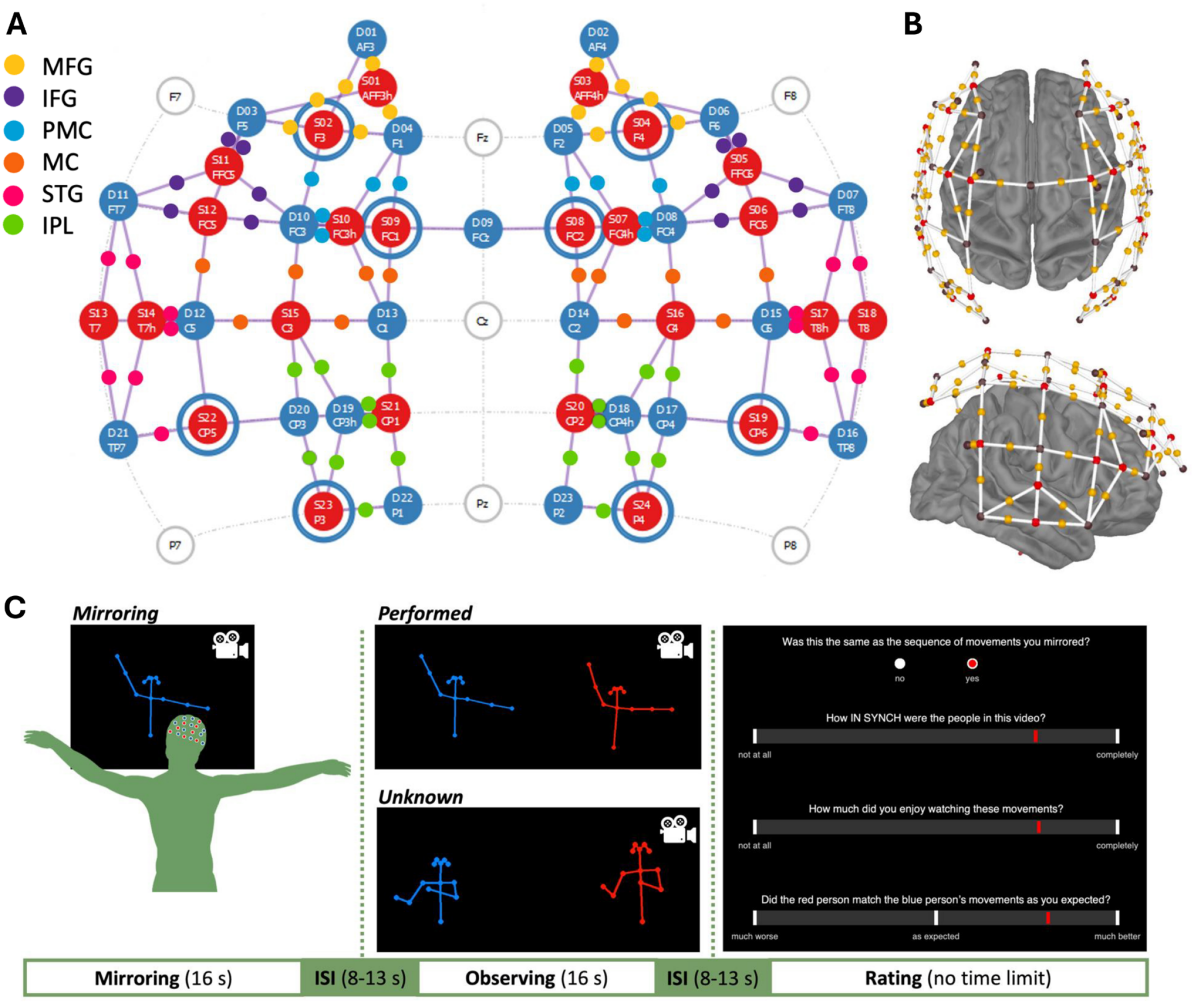
(A) Positions of optodes on scalp according to 10–5 system (Oostenveld & Praamstra, 2001). The montage includes source optodes (red-filled circles), detector optodes (blue-filled circles), short-channel detector optodes (blue rings around red-filled circles), and channels (grey lines). Channels are colour coded by ROI (smaller dots). MFG = middle frontal gyrus, IFG = inferior frontal gyrus, PMC = premotor cortex, MC = motor cortex, STG = superior temporal gyrus, IPL = inferior parietal lobule. (B) Optode positions visualised over brain with source optodes (red spheres), detector optodes (black spheres), points of measurement (yellow spheres), and channels (white bars). (C) Schematic visualisation of a single trial. Participants’ responses to the question “Did the red person match the blue person’s movements as you expected?” are reported in Moffat and Cross (2024a).

We generated 40 videos of 2 stick figures moving in synchrony from videos of people playing the mirror game for 2.5 min at a time (part of a previous experiment: Moffat et al., 2022). We chose stick figures on a black background instead of original videos to mitigate possible influences of gender, skin tone, and facial expressions on participants’ responses (Macpherson et al., 2020). To generate the videos, we first estimated the body positions per person per frame in the original videos using OpenPose (Cao et al., 2019). We then arranged the coordinates extracted by OpenPose into time series data using code adapted from de Jonge-Hoekstra (https://osf.io/6s73d/), including smoothing with a Savitzky-Golay filter (window length = 13 frames, polynomial order = 2) implemented with the signal R package (version 0.7-7; signal developers, 2014). Next, for every frame, we rendered the stick figures (dots for joints, joined by lines) using the Pillow Python package (Clark, 2015) and joined the frames to create videos using Python package OpenCV (version 4.5.5.62; Bradski, 2000). We then extracted 16-s segments from the 2.5-min videos using ffmpeg (Tomar, 2006), which R.M. and a junior laboratory member screened for motion-capture artefacts. The final set of stimuli encompassed 40 videos (Fig. 1A). In all videos, the blue stick figure on the left led the mirror game while a red stick figure on the right followed. We opted for this fixed colour–position relationship to reduce prediction errors related to the stimulus format and to allow participants to focus on the level of synchrony in the movement sequences.

We randomly divided the 40 videos between the *performed* and *unknown* conditions (20 videos each; conditions described in more detail below). We next made copies of the 20 videos in the performed condition, removing the red stick figure and keeping only the blue stick figure centred in the video (Fig. 1A).

### 2.3 Operationalisation of movement synchrony, estimation error, and complexity

#### 2.3.1 Movement synchrony

For each of the 40 videos, we computed movement synchrony by first creating matrices that represent the poses of each member of a dyad per frame and then comparing the matrices to extract a value representing their similarity. This entailed estimating the Euclidian distance between each dyad member’s joints (all combinations of neck, shoulders, elbows, and hands). The distances were listed in a “pose matrix” per dyad member, where they were normalised (negating any influence of difference in height or position relative to the camera). Next, dyad members’ matrices were compared frame-by-frame, to obtain a value between 0 and 1, where 0 corresponded to no similarity of pose between dyad members and 1 corresponded to identical poses between dyad members. Finally, we averaged across all frames in each video to obtain an objective measure of movement synchrony. For additional details, see previous studies that have implemented this procedure (Broadwell & Tangherlini, 2021; Moffat & Cross, 2024b).

#### 2.3.2 Estimation error

We calculated estimation error for each video by subtracting observers’ estimations of movement synchrony from objectively measured movement synchrony (as described above under “Movement synchrony”). An example: The objectively measured level of synchrony in a video is 0.70 (possible values include 0–1), and an observer estimated the level of synchrony to be 60 (on the scale from 0 to 100). We multiplied the objectively measured movement synchrony by 100 to match the scale of the estimations, then subtracted the estimation (70–60 = 10). Positive values reflect underestimation and negative values reflect overestimation.

#### 2.3.3 Movement complexity

We computed movement complexity using the sample entropy (Richman & Moorman, 2000) function int the R package pracma (version 2.4.2; Borchers, 2022). We computed sample entropy for the right and left wrists of both dyad members on each the x- and y- axis (2 axes*2 hands*2 people). Next, we averaged these eight values per video. Entropy values closer to 0 represent greater “signal purity”, which equates to lower complexity (Richman & Moorman, 2000).

### 2.4 Procedure

After providing written informed consent, participants completed a demographic questionnaire. The questionnaire contained extraversion (Goldberg, 1992), self-esteem (Rosenberg, 1965), body perception (Cabrera et al., 2018), body competence (Miller et al., 1981), empathy (Davis, 1980), and autistic trait scales (English et al., 2021). Next, in the main experiment, participants watched 60 videos of 16 s, presented in 3-video blocks. Within each block, the first video showed a single blue stick figure moving its arms. Participants were instructed to match the figure’s arm movements as closely as possible in time and space (*mirroring* condition). Next, participants watched, without moving, two more videos, each showing two stick figures playing the mirror game. The order of these two videos was randomised, with one showing the same sequence of arm movements (*performed* condition) or a different sequence (*unknown* condition). Participants estimated the level of synchrony in the two stick figures’ movements, rated how much they enjoyed watching the movements and indicated whether they recognised the sequence as the sequence they had mirrored, at the beginning of the same block.

### 2.5 fNIRS equipment

We recorded fNIRS signals using a NIRScoutX (NIRx Medical Technologies LLC) and NIRStar software (version 15.3). The NIRScoutX had 24 LED sources, which emitted wavelengths of 760 and 850 nm, and 32 avalanche photodiode detectors. The sampling rate was 2.6 Hz. We positioned optodes on participants’ heads using mesh caps marked with International 10–5 positions (Oostenveld & Praamstra, 2001). Optode positions were selected using the AAL2 atlas within the fOLD toolbox (Rolls et al., 2015; Tzourio-Mazoyer et al., 2002; Zimeo Morais et al., 2018). We arranged optodes over left and right PFC, IFG, STG, and IPL in a montage of 24 sources, 23 detectors, and 8 short detectors (Fig. 1, B and C). This optode arrangement included 78 long channels (~30 mm inter-optode distance, which is adequate for recording changes in blood oxygenation in cortical tissue but also contains signal from the scalp and skull) and 8 short channels (8 mm inter-optode distance, which record changes in blood oxygenation in the scalp and skull and can be used to isolate the cortical signals). We distributed the short channels across the ROIs, taking into account the spatial heterogeneity inherent to extracerebral signals (Brigadoi & Cooper, 2015; Gagnon et al., 2012; Zhang et al., 2015),

### 2.6 Data analysis

We performed our statistical analyses in the R language (version 4.3.1; R Core Team, 2022) within the RStudio IDE (version 2023.06.1; RStudio Team, 2020). We fit Bayesian multilevel models using the brms package (version 2.20.1; Bürkner, 2017) and computed parameter estimates and contrasts using the emmeans package (version 1.8.8; Lenth, 2021). For ease of interpretation and comparison, we homogenised the scale of predictors (i.e., traits and kinematic measures) using Z-scores. We defined weakly informed priors for our models, thereby imposing a constrained distribution on our expected results. In doing so, we drew on existing knowledge regarding the possible outcomes in a way that allows for plausible large effects and for the data to dominate the structure of the posterior distribution (Gelman, 2006; Lemoine, 2019). We report and interpret the posterior distributions of parameters with a 95% credible interval, computed using the highest posterior density region (HPD) method (McElreath, 2020). Following this approach, p-values are not calculated and correction for multiple comparisons is not required. For readers accustomed to a Frequentist approach and interested in gaining familiarity with the Bayesian approach, we recommend perusing Kruschke & Liddell (2018).

#### 2.6.1 fNIRS

Analyses were performed as described in Moffat & Cross (2024a). That is, we used Python packages MNE (version 1.4.2; Gramfort et al., 2013), MNE-NIRS (version 0.5.0; Luke et al., 2021), NiLearn (version 10.1; Abraham et al., 2014), and statsmodels (version 0.14.0; Seabold & Perktold, 2010) to estimate the amplitude of evoked haemodynamic responses per ROI and condition via a generalised linear model (GLM) approach (Huppert, 2016). We took the following preprocessing steps: (i) converting the signal from raw intensity to optical density, (ii) correcting motion artefacts using the temporal derivative distribution repair algorithm (Santosa et al., 2019), and (iii) converting the signal from optical density to concentrations of oxygenated haemoglobin (HbO) and deoxygenated haemoglobin (HbR) using the Modified Beer–Lambert Law (Delpy et al., 1988; Kocsis et al., 2006). We used a partial pathlength factor of 0.1, which accounts for the differential pathlength factor (DPF) and partial volume correction (PVC), where (DPF = 6)/(PVC = 60) is equal to 0.1 (Santosa et al., 2018; Strangman et al., 2003). Visualisations of estimated haemodynamic response amplitudes are included in Supplementary Figures 1-3.

We fit the GLM to the long channels (>20 and <40 mm, which probe to the depth ~15 mm, recording signals from scalp, skull, and cortex). No channels were excluded for any participant (see visualisation of signal quality, i.e., scalp-coupling index (Pollonini et al., 2014), per ROI and participant in Supplementary Fig. 5). To construct the design matrix, a 16-s boxcar function at each event-onset-time was convolved with the canonical haemodynamic response function (Glover, 1999; Santosa et al., 2019). The principal components of short channels (<20 mm, which primarily record signals from tissue superficially located relative to the cortex) and drift orders up to 0.01 Hz were included in the GLM (Huppert, 2016). The GLM was performed with a lag-1 autoregressive noise model, to account for the correlated nature of the fNIRS signal components. We averaged estimates for each participant within each ROI, weighted by the standard error of the per-channel estimates for that ROI.

We then calculated the HbO-HbR difference by subtracting HbR from HbO estimates per participant, ROI, and condition. The HbO-HbR difference (sometimes called HbDiff) measure offers the advantage that the sign (+/-) indicates whether the measured response is canonically oriented (+) or inverted (-). Inverted responses are also called negative BOLD response. Moreover, each HbO-HbR difference value can be categorised by the relationship between the original estimates: Taking a conservative stance, HbO-HbR differences from the same sign (HbO and HbR both + or both -) may reflect blood pressure or extracerebral changes rather than true cortical activation, where HbO and HbR have opposite sign (Wolf et al., 2002; Yücel et al., 2021; Zimeo Morais et al., 2017). Retaining HbO-HbR differences calculated from estimates with opposite signs can thus be considered a conservative approach. HbO-HbR difference is commonly used in fNIRS studies examining clinical and cognitive phenomena and is well placed to enhance modelling of complicated relationships between cortical activation and behaviour (Corp et al., 2018; Hakim et al., 2022; Kaynezhad et al., 2019; Kolyva et al., 2014; Moffat et al., 2022, 2023; Moffat & Cross, 2024a). To address our group-level hypotheses pertaining to cortical activation, we employed Bayesian multilevel Gaussian models as described above.

## 3 Results

### 3.1 Influence of embodiment on estimation of movement synchrony

**Fig. 2.**
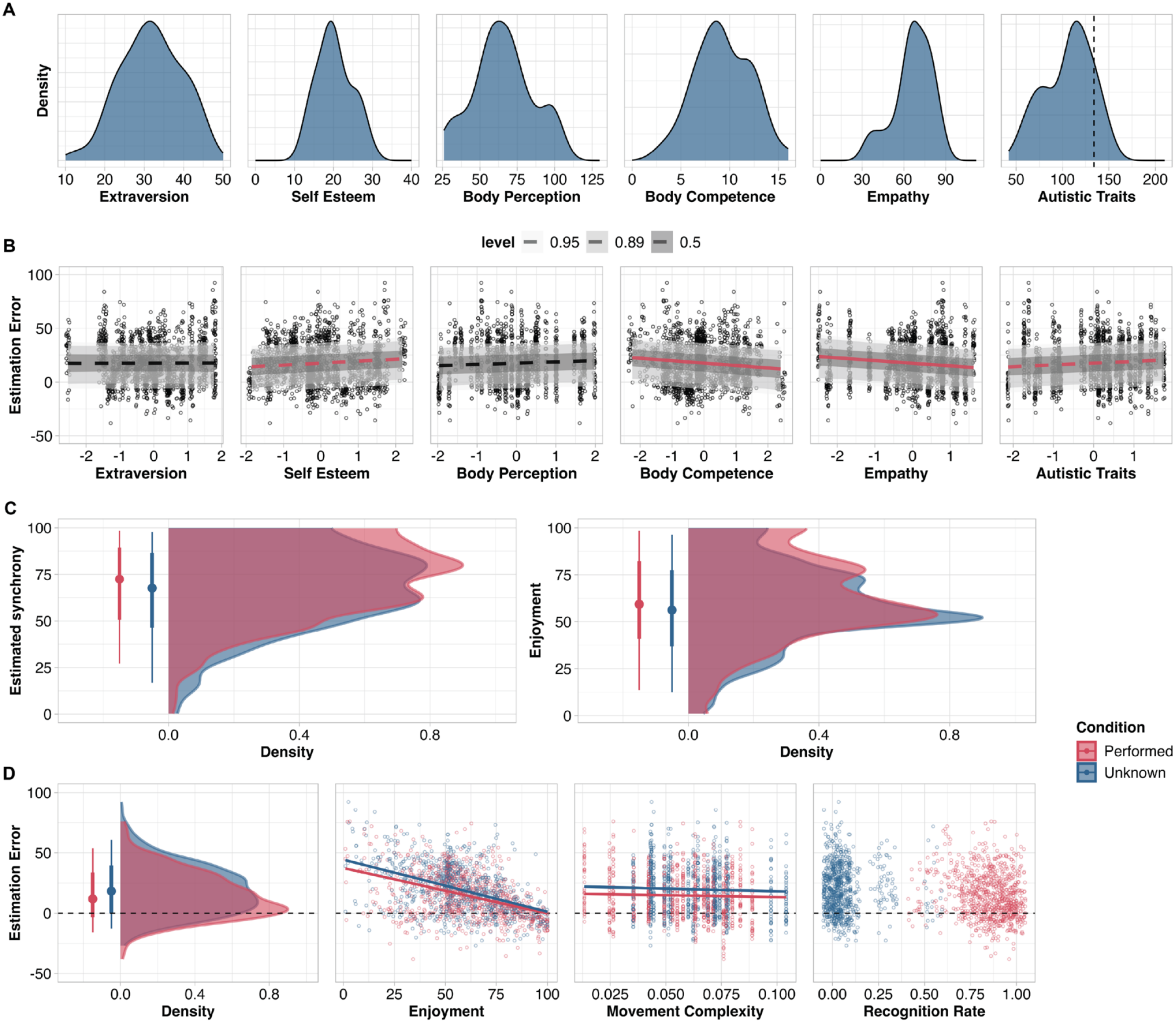
(A) Distributions of scores on individual measures (in raw units). Density axis left blank to scale distributions according to maximum values. For autistic traits, the dotted vertical line indicates the threshold above which scores are likely to reflect a clinical diagnosis of an autism spectrum disorder. Each measure was z-scored before being included in models. (B) Predicted association between interindividual traits and participants’ estimation error when estimating levels of synchrony. Solid red lines show substantial trait–estimation error associations (HPD do not overlap with 0); dashed red lines show trends, and dashed black lines show non-substantial associations. Shading shows 95%, 89%, and 50% intervals of the posterior predictive distribution per parameter. (C) Distributions of estimated movement synchrony levels and enjoyment ratings (both in raw units between 0 and 100). Points and bars show mean and intervals covering 66% and 99% of the full distribution. (D) Distributions of estimation error and associations between estimation error and each ratings of enjoyment, movement complexity, and recognition of movement sequences. Points and bars in left-most panel show mean and intervals covering 66% and 99% of the full distribution. See Supplementary Figure 1 for visualisation of estimated synchrony levels as a function of measure synchrony levels.

Distributions of each interindividual trait are visualised in Figure 2A. Though slightly broader, these distributions are consistent with those in our previous work on interindividual differences in a separate sample of 322 participants (Moffat & Cross, 2024b). Following the characterisation of the sample, we assessed the associations between interindividual traits and estimation of movement synchrony using the model defined in the previous section. Distributions of estimated synchrony levels and enjoyment ratings are presented in Figure 2C, and Figure 2D for estimation error. See Supplementary Table 1 for descriptive statistic for all measures.

We assessed the influence of condition (performed vs. unknown) using the model that included personality traits: *measured-estimated synchrony ~ 1* *+* extraversion * condition *+* self-esteem * condition *+* body perception * condition *+* body competence * condition *+* empathy * condition *+* autistic traits * condition *+* *(1|ID)*.

Observers underestimated synchrony for performed and unknown movements (*performed: ß* = 14.40, HPD = [12.10, 16.80]; *unknown: ß* = 19.10, HPD = [16.80, 21.40]; Fig. 2D). Contrasts reveal that participants’ underestimation of synchrony was more pronounced for unknown than performed movements (*ß* = -4.72, HPD = [-5.93, -3.55]).

### 3.2 Replication: Estimation of movement synchrony with respect to interindividual trait and rating differences

Body competence scores (*ß* = -2.27, HPD = [-4.36, -0.05]) and empathy scores (*ß* = -2.25, HPD = [-4.42, -0.09)] were negatively associated with estimation error (higher scores co-occurred with less underestimation of movement synchrony; Fig. 2B). Self-esteem scores (*ß* = 1.76 HPD = [-0.44, 3.99]) and number of autistic traits (*ß* = 1.66, HPD = [-0.83, 3.98]) showed trending associations, whereby higher scores co-occurred with greater underestimation of movement synchrony. Extraversion scores (*ß* = -0.10, HPD = [-2.25, 2.14]) and body perception scores (*ß* = 1.16, HPD = [-1.02, 3.27]) were not associated with estimation error. We observed no differences for any trait when comparing the slopes of estimation error–trait associations between conditions (i.e., performed/unknown).

To shed light on the extent to which the current work replicates our previous work (Moffat & Cross, 2024b), we examined the relationships between movement complexity, participants’ ratings of enjoyment, and participants’ recognition of movement sequences as being performed or unknown (Fig. 2D). Enjoyment was negatively associated with estimation error, that is, lower ratings of enjoyment are associated with greater underestimation of synchrony (*ß* = -0.40, HPD = [-0.46, -0.36]). We observed no evidence for a substantial difference in the strength of the estimation error–enjoyment association between unknown and performed movements (*ß* = 0.052, HPD = [-0.01, 0.11]). Movement complexity was negatively associated with estimation error (*ß* = -35.60, HPD = [-54.70, -16.70]), whereby observers tended to estimate synchrony levels with less error (i.e., underestimate less) for more complex sequences of movements. Contrasts revealed no substantial difference in the strength of the estimation error–movement predictability association between performed and unknown movements (*ß* = 12.70, HPD = [-5.49, 31.80]). With respect to recognition of movement sequences, we observed no substantial association between estimation error and participants’ recognition of sequences as performed or unknown (*ß* = -2.96, HPD = [-10.60, 4.77]).

### 3.3 Estimation of movement synchrony and associated patterns of cortical activation

We have reported the cortical activation of AON regions while participants viewed performed and unknown movement sequences in a previous publication (Moffat & Cross, 2024a). Visualisations of estimated haemodynamic response amplitudes are included in Supplementary Figures 2-4. In our previous work, we found that participants’ awareness of having performed a movement sequence and enjoyment of movement sequences to be associated with the amplitude of cortical responses while viewing synchronous movements. Thus, in the present study, we accounted for recognition and enjoyment in our model predicting the relationship between participants’ abilities to estimate synchrony levels and cortical activation in AON regions: *HbO-HbR ~ 1* *+* Condition * ROI * mean measured-estimated synchrony *+ recognition rate* *+* *mean of enjoyment ratings* *+* *(1|ID)*.

For performed and unknown conditions aggregated, we observed trends of a negative association between estimation error and activation over for both left and right IPL (“Aggregated: Performed + Unknown” in Table 1).

**Table 1.** Parameter estimates describing the associations between estimation error and cortical activation for each ROI, for both conditions combined (left), and for conditions contrasted against each other (right).

	Aggregated: performed+unknown		Contrast: performed-unknown	
ROI	Estimate	HPD	Estimate	HPD
LIFG	-0.02	-0.12, 0.09	**-0.21**	**-0.33, -0.09**
LMFG	-0.01	-0.11, 0.10	**-0.17**	**-0.30, -0.03**
LPMC	-0.06	-0.17, 0.04	-0.08	-0.22, 0.05
LMC	-0.05	-0.16, 0.04	*-0.10*	*-0.23, 0.02*
LSTG	-0.08	-0.21, 0.04	**-0.21**	**-0.38, -0.05**
LIPL	*-0.09*	*-0.19, 0.01*	-0.06	-0.19, 0.07
RIFG	-0.02	-0.13, 0.09	-0.04	-0.19, 0.10
RMFG	-0.05	-0.16, 0.05	*-0.10*	*-0.23, 0.02*
RPMC	-0.03	-0.14, 0.07	-0.07	-0.21, 0.06
RMC	-0.06	-0.17, 0.05	**-0.15**	**-0.28, -0.01**
RSTG	-0.05	-0.17, 0.07	-0.11	-0.28, 0.05
RIPL	*-0.10*	*-0.21, 0.00*	-0.10	-0.24, 0.03

Bold indicates substantial associations and contrasts where the HPD does not overlap with 0. Italics indicate trends towards substantial associations and contrasts where <10% of HPD overlaps with 0.

**Fig. 3.**
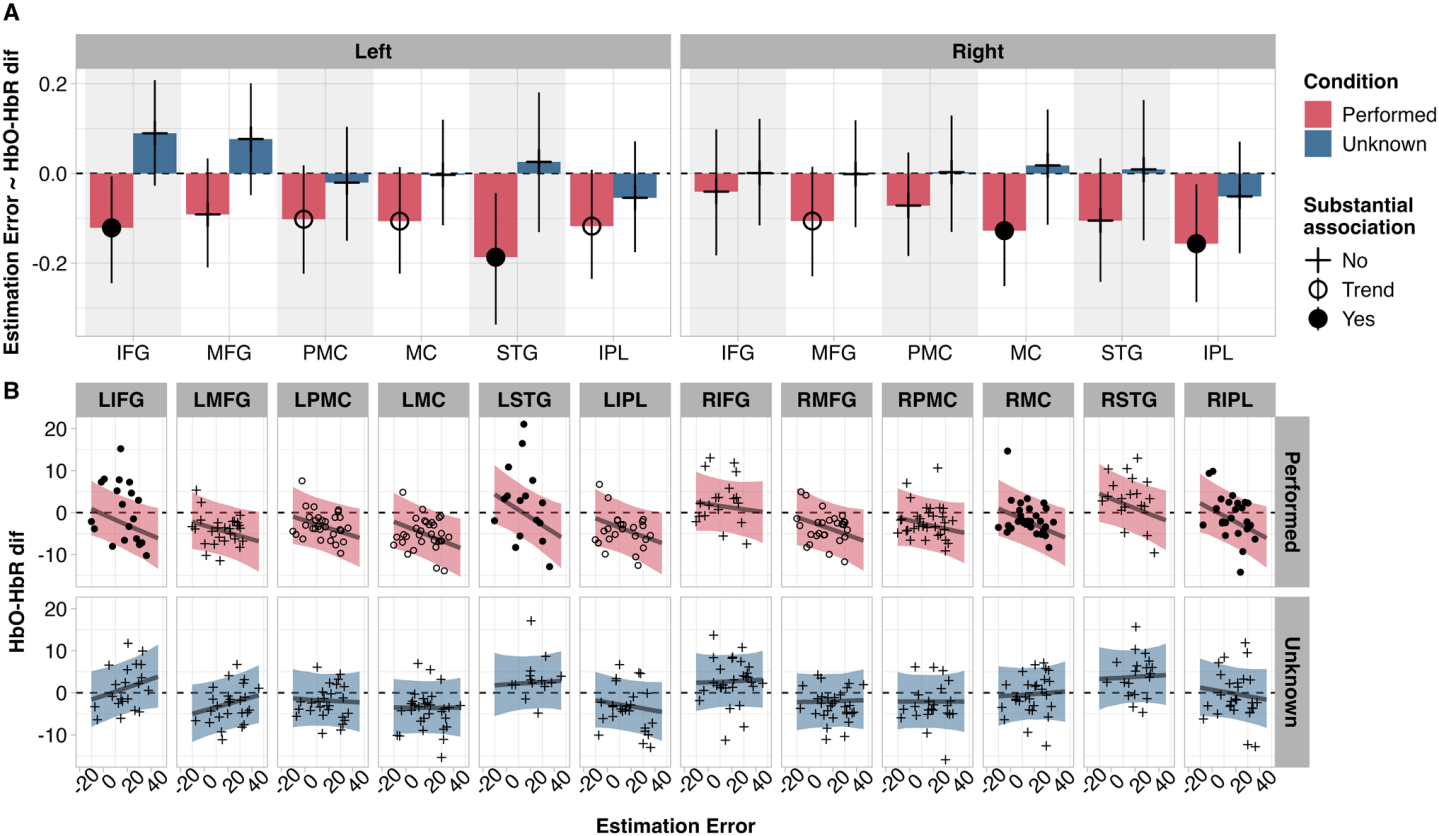
(A) Visualisation of parameter estimates for the associations between estimation error and cortical activation in each ROI. In other words, each bar represents a slope, for example, over left IFG (LIFG), the red bar for Performed indicates a substantial negative association between cortical activation and estimation error. Error bars show HPD. (B) Raw data plotted on top of line showing the predicted association between estimation error and cortical activation. Shading shows 95% intervals of the posterior predictive distribution.

Planned contrasts between performed and known movement sequences highlighted the prevalence of negative associations specifically for performed movements (red bars in Fig. 3). Activation in left IFG and STG, as well as right MC and IPL, was negatively associated with estimation error for performed movements, while left PMC, MC, and IPL, as well as right MFG, showed trending negative associations. Participants who underestimated synchrony levels the most showed inverted haemodynamic responses, whereas participants who estimated correctly showed responses closer to zero or positive responses. For unknown movements, no such associations were observed.

Next, we contrasted the estimates for the association between estimation error and cortical activation per ROI (“Contrast: Performed-Unknown” in Table 1). For performed relative to unknown movements, stronger negative associations between cortical activation and measured-estimated difference were observed for left IFG, MFG, STG, and with a matching trend for left and right MC and MC.

### 3.4 Exploring role of body competence in brain–behaviour relationships

We conducted a further exploratory analysis of the association between estimation error and cortical activation for three levels of body competence scores (1 SD below mean, mean, 1 SD above mean) to outline a possible step future work could take and to elucidate the role of embodied experience in the negative association between estimation error and cortical activation while observing performed movements. We observed that the negative associations between estimation error and cortical activation were substantial at 1 SD above the mean body competence score, and not substantial at the mean or 1 SD below the mean (see Supplementary Fig. 6). Comparisons between SD levels revealed no substantial differences in the strength of the three negative associations. Given the small sample size and the exploratory nature of this extra analysis, these findings should be interpreted with caution.

## 4 Discussion

In this study, we set out with three aims. First, we aimed to establish the extent to which embodied movement experience altered individuals’ abilities to estimate levels of movement synchrony. We found that embodied experience led participants to estimate synchrony with less error (i.e., less underestimation) when observing movements they had performed, relative to unknown movements. Second, we aimed to extend our previous findings regarding the role of embodied movement experience in shaping AON responses (Moffat & Cross, 2024a) by examining the degree to which individuals’ ability to estimate synchrony levels is reflected in cortical responses to observed synchronous movements. Differences in estimation error predicted cortical activation measured over left IFG and STG, and right MC and IPL for performed movements, while no ROIs were associated with differences in estimation error for unknown movements. Third, we aimed to examine the relationship between estimation error and individual personality traits, as well as enjoyment and movement complexity, to replicate our previous findings (Moffat & Cross, 2024b). We observed that greater body competence and empathy were associated with less underestimation (less error). We also found trends for greater self-esteem and more numerous autistic traits to be associated with greater underestimation (more error). Moreover, greater enjoyment of movement sequences and lower movement complexity were associated with greater underestimation, while explicit recognition of movement sequences did not influence estimation of synchrony levels. In the following, we unpack the implications of each of these findings in turn.

### 4.1 Embodied movement experience improves estimation of synchrony levels

A wealth of evidence suggests that embodied experience gained through physical movement enables the human motor system to simulate previously performed body movements without initiating physical movement. This evidence base further suggests that the fluency of such motor simulations shapes observers’ perception of observed body movements (e.g., Cross & Orlandi, 2020; Gallese et al., 2018; Keysers et al., 2018). In the present study, participants acquired embodied experience by practicing specific movement sequences immediately before viewing these same movement sequences performed by two other people. Their task was to then estimate the level of synchrony in those people’s movements. In doing so, we captured how embodied movement experience influences observers’ abilities to estimate levels of synchrony. We found that observers estimated levels of synchrony with less error for movement sequences that they had physically performed than sequences they had not performed. Participants’ estimation error decreased as dyadic movements became more complex, regardless of whether participants had performed the movement or not, suggesting that the relationship between movement complexity and synchrony estimation is not sensitive to embodied experience. Moreover, participants’ estimation error was not impacted by their awareness of having performed the movements (as measured by correct recognition). We take these findings as evidence that that embodied movement experience heightens perceptual sensitivity to movement synchrony, which we speculate may be related to having established the neural basis for motor simulation of the movements.

The notion of heightened perceptual sensitivity to movement synchrony following bodily experience aligns with other forms of enhanced perceptual sensitivity to the movement kinematics (performed by single people), resulting from embodied movement experience. In the field of sports science, researchers have examined motor expertise-based differences in perceptual sensitivity to subtle kinematic cues spanning a multitude of physical activities including gymnastics, tennis, and martial arts (e.g., Giblin et al., 2016; Mack, 2019; Papesh et al., 2021; Vinken & Heinen, 2021). Such studies have revealed that embodied experience facilitates anticipation of the outcomes of movements, as well as adjudication of the “correctness” of movement skills. By asking our participants to estimate the level of movement synchrony, we effectively positioned our participants as judges of a certain type of “correctness” of movement synchrony rather than a specialised sporting skill. Embodied experience enhanced participants’ estimation of movement synchrony levels, demonstrating that the perceptual benefit extends beyond single person movement, at least to dyadic movements. These findings reinforce the importance of physical practice for acquiring the perceptual skills needed to guide other people in learning synchronous endeavours. That is, coaches and judges of athletes, performers, or enthusiasts who strive to achieve movement synchrony (e.g., synchronised figure skaters and divers, rhythmic gymnasts, Zumba or line dancing enthusiasts) are most likely to build their perceptual sensitivity to levels of movement synchrony through physical practice. The degree to which the ability to perceive synchrony is sport/activity-specific remains an open question.

### 4.2 Individual differences in sensitivity to synchrony levels shape AON responses

Embodied experience is believed to facilitate motor simulation processes that occur when we watch another person move their body (Cross & Orlandi, 2020; Gallese et al., 2018; Keysers et al., 2018). Arguably, the most direct form of evidence for motor simulation has been established by using TMS to disrupt and quantify motor resonance (as described in Section 1.2). Relative to TMS stimulation paradigms, our brain imaging technique and experimental design offer less direct insight into the strength of observers’ motor simulation per se. Nonetheless, we carefully implemented our design to explore the link between individuals’ AON activation (a less direct proxy for motor simulation) and individuals’ abilities to estimate synchrony levels for physically performed versus unknown, unperformed sequences. We were surprised by the striking difference between performed and unknown movement sequences, in terms of the strength of the association between cortical activation and estimation error. For unknown movements, no AON regions showed any associations with estimation error. For performed movements, four bilaterally distributed AON regions (left IFG and STG, and right MC and IPL) showed substantial negative associations with estimation error that differed substantially from the associations for unknown movements, with similar trends in left PMC, MC, and IPL, and right MFG. The bilateral distribution of these regions aligns with previous reports of the neural correlates of synchrony observation (Cross et al., 2024; Orgs et al., 2024)—though the negative association with estimation error for performed movements remains novel. We propose that the presence of these associations for performed movements and absence thereof for unknown movements could be suggestive of different levels of motor simulation fluency, which in turn aligns with the observed reductions in estimation error.

#### 4.2.1 Motor simulation as a potential mechanism underpinning synchrony estimation

Research spanning neuroimaging modalities has demonstrated that motor simulation is associated with patterns of increased activation in motor regions that closely resemble the patterns of activation evoked by physical movement (Gallese et al., 2018; Keysers et al., 2018). By this account, embodied motor experience gained in the performed condition should have resulted in increased AON activation when observing performed, as opposed to unknown, sequences. Our previously published analysis showed no substantial differences for performed or unknown sequences in any ROIs which we attributed to the brevity of the embodied experience—one repetition of a 16-s long movement sequence (Moffat & Cross, 2024a). In this task involving complex dyadic movements, as opposed to simpler and more constrained unimanual movements, participants’ individual profiles of cumulative embodied experience likely shape how fluently their motor systems encode and simulate the movements they performed once. Indeed, our findings that left IFG and STG, and right MC and IPL are selectively sensitive to estimation error for performed movements lend support to the interpretation that participants’ motor systems may have encoded representations of the performed movement sequence with varying degrees of success/fidelity and that the variability in these representations may carry through to participants’ accuracy in estimation of synchrony levels. We thus propose that individual differences at the encoding stage are likely to be explained by cumulative embodied experience (Cross & Orlandi, 2020; Orgs et al., 2024; Orlandi et al., 2020). To disentangle the contribution of cumulative embodiment from motor experience gained in our experimental paradigm, future studies must account for individual differences in cumulative embodiment (as modelled by our preliminary analysis of the relationship between body competence, synchrony estimation, and cortical activation in AON regions reported in Section 3.4).

Shedding light on embodied experience and movement synchrony, a recent fMRI study compared the similarity of expert and non-dancers’ patterns of brain activity (i.e., intersubject correlation; ISC) while they watched videos of 10-person dance performances (Orgs et al., 2024). The authors report that movement synchrony among the 10 dancers predicted approximately 10% changes in ISC among expert and non-dancers, particularly in PMC, and MC, dmPFC, as well as orbitofrontal and cingulate cortices. However, for non-dancers but not experts, a portion of ISC was also predicted by the amount of movement of dancers. These findings demonstrate that the experts’ (with a wealth of embodied experience) brains represent movement synchrony similarly. This was observed to a lesser degree among novices, lending strong support to the idea that embodied experience alters the behavioural and neural mechanisms underpinning perceptions of movement synchrony (Orgs et al., 2024). We could imagine extending on this experimentally rigorous work to investigate the relationship between expert/non-dancers’ abilities to estimate synchrony levels and ISC, to better understand the dynamic time course of synchrony perception.

We noted that participants who underestimated synchrony levels tended to show inverted haemodynamic responses while viewing performed movements (less so for unknown movements). This was numerically true across all AON ROIs, though only statistically substantiated for left IFG and STG, and right MC and IPL (Fig. 3B). The origin of inverted/negative responses recorded using fNIRS and fMRI remains a topic of debate (He et al., 2022; Maggioni et al., 2016; Mullinger et al., 2014). Emerging evidence suggests inverted haemodynamic responses could be useful for characterising brain activity during cognitive tasks, on the basis that inverted responses characteristics are more variable and task specific (Gholipour Picha et al., 2024). We speculate with the utmost caution that co-occurrences of inverted haemodynamic responses and poor estimation of synchrony levels could reflect individual-specific differences in working memory capacity (S. Liu et al., 2018; Newton et al., 2011). A theoretical model of working memory proposed by Christophel et al. (2017) involves a distributed network of prefrontal, parietal, and sensory brain regions that differentially process low-level, concrete and higher-level, abstract information. Applying Christophel’s theoretical model of working memory to perception of dyadic movement synchrony, “low-level, concrete” could refer to the physical positions of each persons’ right hand, and “high-level/abstracted” could refer to how hand-position information is altered to predict upcoming movements (Christophel et al., 2017). From this perspective, it is possible that differences in working memory while observing and estimating movement synchrony may be reflected in the AON regions that we observed to be sensitive to estimation error. Future research could examine whether negative bold responses are related to working memory capacity when observers view synchronous movements, to test this tentative hypothesis.

### 4.3 Replication of existing findings

Previous research has established that observers tend to underestimate synchrony levels in multi-person movement sequences overall and that individual differences in traits and aesthetic experiences constrain the degree to which individuals underestimate synchrony (Moffat & Cross, 2024b; Vicary et al., 2017). With respect to individual traits, we found that body competence and empathy scores were associated with less underestimation, and that greater self-esteem and autistic traits trended towards being associated with greater underestimation. Of these four relationships between traits and estimation error, two replicate the findings of our previous work (Moffat & Cross, 2024b). That is, in a separate sample of 322 observers with no embodied experience of the movements, we observed that greater body competence scores predicted reduced underestimation and that more numerous autistic traits predicted increased underestimation of movement synchrony. The present study also replicated our prior finding that greater enjoyment while observing dyadic movements was associated with reduced underestimation (i.e., reduced estimation error), as was greater movement complexity (referred to as predictability in our previous work). The replication of these four relationships (two traits, one aesthetic judgement, and one kinematic measure) bolsters our confidence in the reliability of these relationships and lays the groundwork for future studies deepening our understanding of individual differences in synchrony estimation.

### 4.4 Limitations

A limitation of this work and other existing studies is the focus on synchrony vs. asynchrony (e.g., Cracco et al., 2022; Cross et al., 2024; Georgescu et al., 2014). In the case of the present study, this is due to the block design (i.e., grouping all performed and unknown trials to calculate haemodynamic response amplitudes). Emerging research documents the neural responses to naturally occurring levels of synchrony uses real-time, continuous dance performances and with real audiences as observers to overcome this limitation (e.g., Orgs et al., 2024; Rai et al., 2025). Future studies, both in the laboratory and in the real world, could employ event-based designs to characterise the neural responses to a wider variety of naturally occurring levels of movement synchrony. Due to the study design, it is not possible to confirm that visual familiarity alone, or in combination with bodily familiarity, was not a contributing factor of the behavioural and cortical response patterns. Future research could disentangle these components by including a condition where movements are viewed but not performed, to generate purely visual familiarity. Moreover, future research should contrast between other networks (e.g., the Default Mode Network) and the AON to pinpoint the specificity of the AON’s involvement in estimation of movement synchrony. Another limitation is that participants’ physical performance (i.e., mirroring) of movements was not recorded, meaning that individual differences in mirroring ability cannot be used to explore differences in estimation of movement synchrony.

## 5 Conclusions

This study explored the extent to which individuals’ abilities to estimate levels of dyadic movement synchrony are reflected in patterns of cortical activation for physically practiced and unknown sequences of movements. We found that observers tend to underestimate synchrony levels, more so for unknown than for performed sequences. Associations between estimation error and cortical activation were observed for performed sequences only. Greater cortical activation in left IFG and STG, and right MC and IPL co-occurred with more accurate synchrony estimation, potentially reflecting more fluent motor simulation of the practiced movements. Moreover, in a successful replication of previous work, we found that embodied experience, personality traits, and ratings of enjoyment predicted how accurately observers estimate levels of movement synchrony. These findings inform our understanding of the role of bodily experience in discerning levels of movement synchrony—a skill that is deeply valuable in leisure and professional sports, as well as the performing arts.

## Supplementary Material

Supplementary Material

## Data Availability

These data were collected after the submission of the following preregistration: https://osf.io/2jn73. The preregistration states that the analyses presented here are envisaged. The full original dataset associated with the preregistration can be found in the OSF repository titled “Embodying aesthetic movement preferences” (https://osf.io/e7w4v/). The portion of the data analysed in this study can be found in our OSF repository entitled “Estimating movement synchrony with and without embodied experience” (https://osf.io/wh2m3/).
