# Supplementary material for: Individual abilities to estimate levels of movement synchrony predict action observation network activation
Source: Imaging Neurosci (Camb). 2025 Oct 24;3:IMAG.a.962. doi: 10.1162/IMAG.a.962 (PMC12556691; doi:10.1162/IMAG.a.962)
Supplement: Supplementary Material [file IMAG.a.962_supp.pdf]

Table 1. Summary of descriptive statistics for all variables included in analysis.

Variable	Mean	Std. Dev.	Min	Max
**Estimated synchrony levels**	68	20	0.21	100
**Enjoyment ratings**	59	21	1	100
**Objectively measure synchrony ([raw value between 0-1] *100)**	85	8.8	59	96
**Complexity**	0.06	0.02	0.013	0.1
**Estimation error** (Estimated synchrony - measured synchrony)	17	20	-38	92
**Extraversion**	32	7.4	13	45
**Self esteem**	20	4.5	12	30
**Body perception**	65	20	27	104
**Body Competence**	9.3	2.8	3	16
**Empathy**	66	13	33	88
**Autistic traits**	106	25	52	150

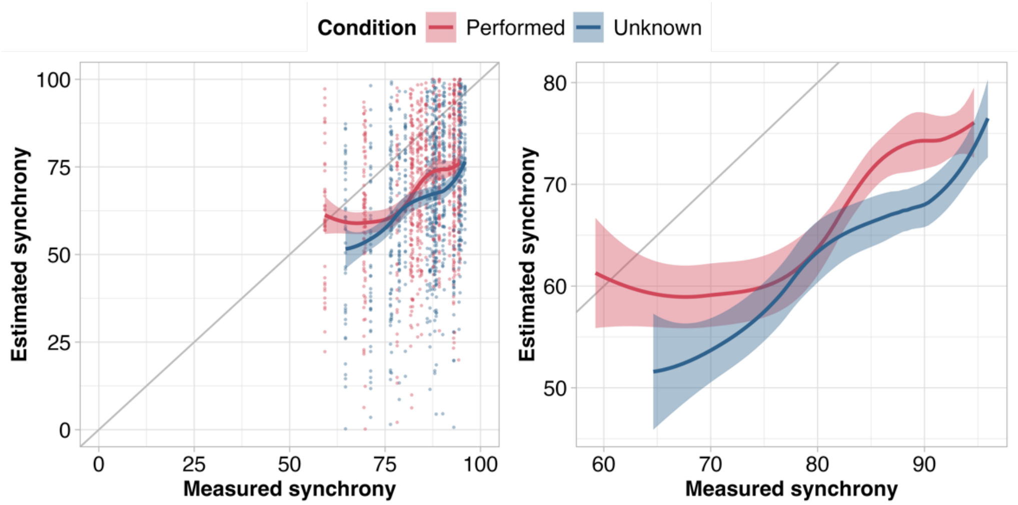

Figure 1. Raw data plotted in the style of a psychophysical curve (measured value on x-axis, participant response on y-axis). The regression lines were plotted using loess (i.e., local) smoothing to visually illustrate potential non-linear relationships. The left panel includes the whole plausible range of values from 0-100 with points per observation. The right panel zooms in on values above 50 to facilitate evaluation of the presented data. The diagonal grey line shows a perfect match between measured and estimated synchrony levels.

Figure 2, Figure 3, and Figure 4 were original published in the supplementary materials for Moffat and Cross (2024), titled “Awareness of embodiment enhances enjoyment and engages sensorimotor cortices”. We present the visualisations here, as the analyses in the present work are performed with the same fNIRS data and these visualisations provide meaningful context.

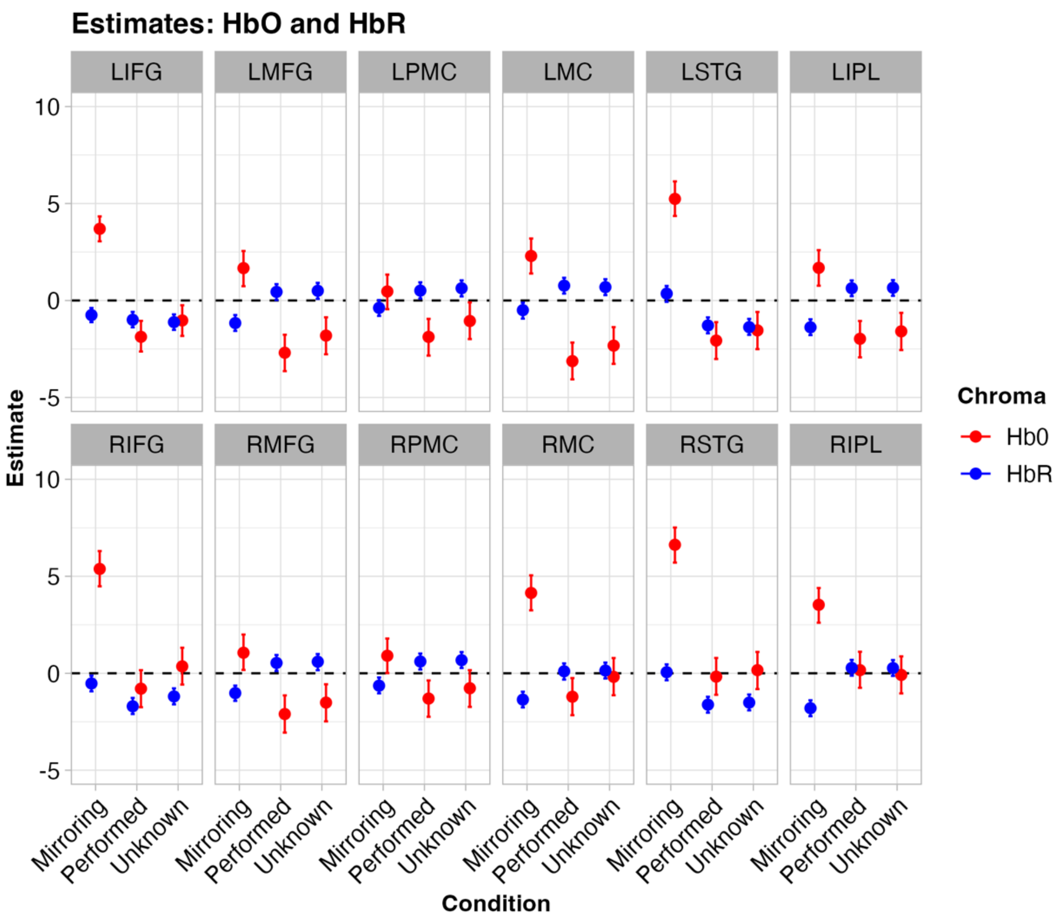

Figure 2. Estimated amplitude of haemodynamic response amplitude for HbO and HbR per condition and ROI. Error bars show HPD. HPD = 95% highest posterior density region.

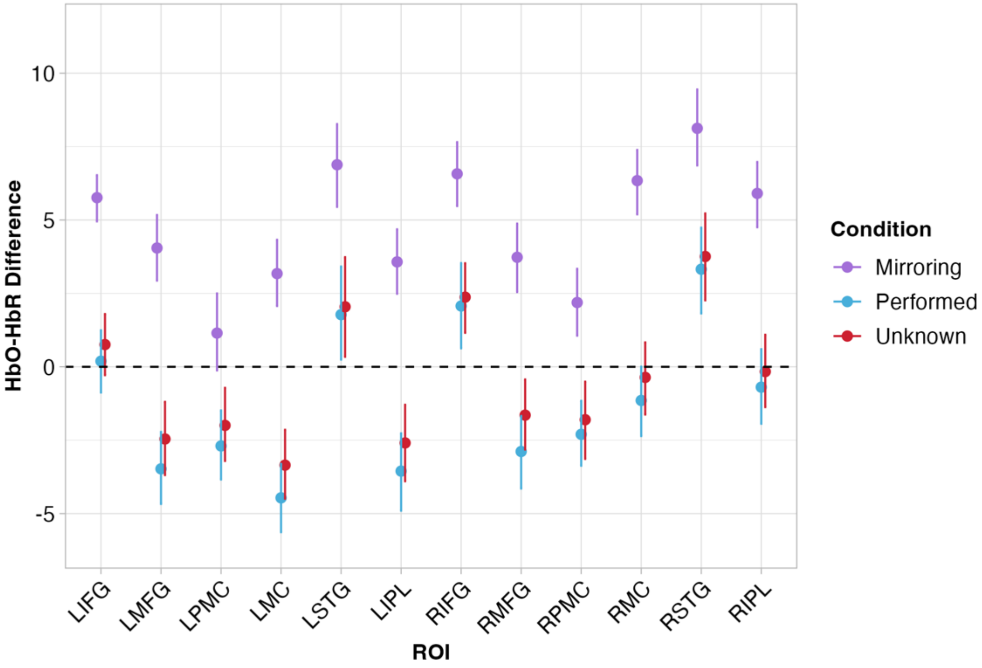

Figure 3. Estimates of negatively correlated HbO-HbR differences per ROI and condition. Error bars show HPD. HPD = 95% highest posterior density region.

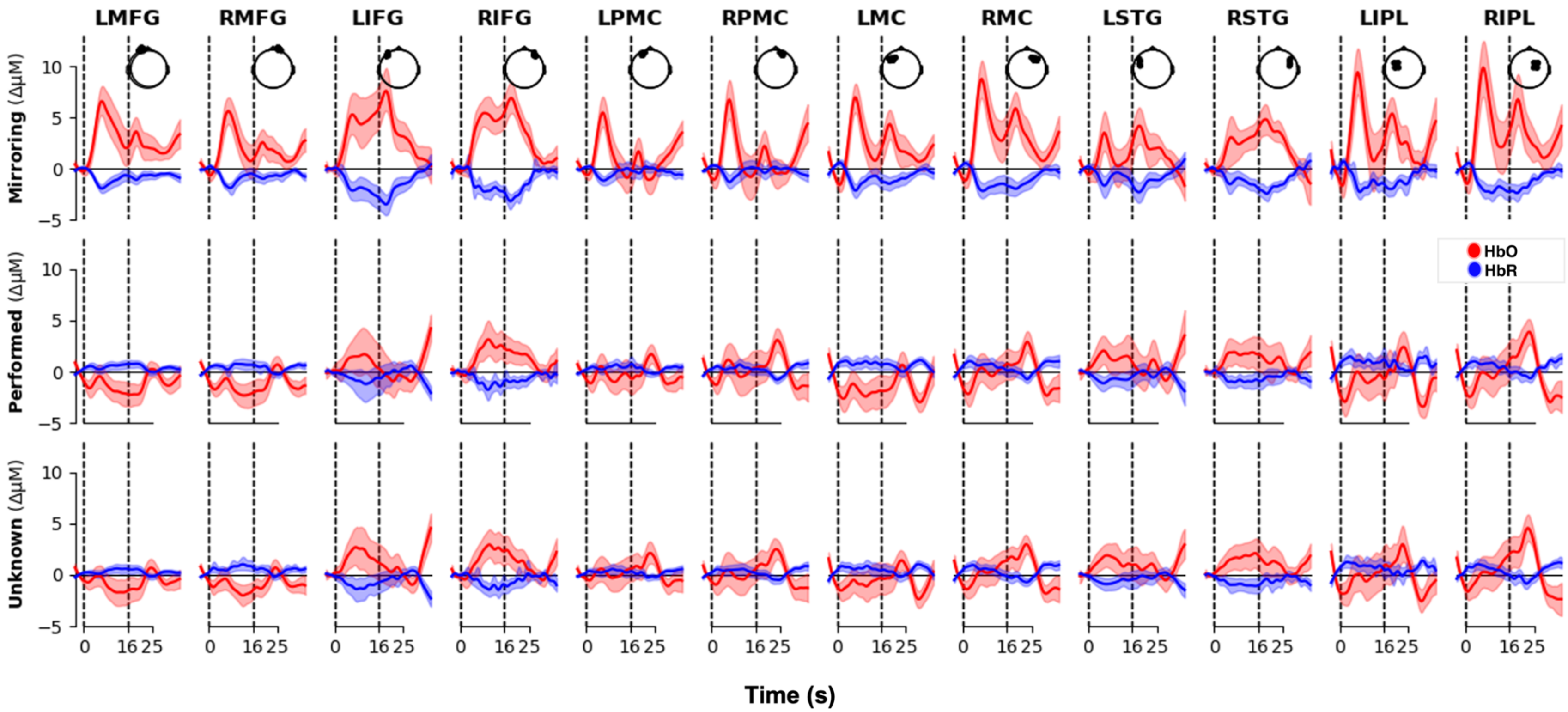

Figure 4. Grand average waveforms per condition and ROI. These were generated for visual inspection of the fNIRS data. Raw intensity was converted to optical density. As a measure of signal quality, a scalp-coupling index (Pollonini et al., 2014), was calculated. Channels with values <0.5 were rejected. Motion artefacts were corrected using the temporal derivative distribution repair (TDDR) algorithm (Fishburn et al., 2019). Next, short-channel regression was applied using the nearest short channel for each long channel, effectively isolating the cerebral signal component by regressing out extracerebral and systemic components (Saager & Berger, 2005; Scholkmann et al., 2014). The signal was then converted from optical density to concentrations of HbO and HbR using the Modified Beer-Lambert Law (Delpy et al., 1988; Kocsis et al., 2006) with a partial pathlength factor of 0.1. Next, we applied Cui et al.'s (2010) algorithm to improve signal-to-noise ratio based on the negatively correlated dynamics of HbO and HbR. The signal was then bandpass-filtered between 0.01–0.3 Hz to exclude slow drifts and cardiac components. Response epochs were trimmed from 5 s before stimulus onset to 30 s post-onset and linearly detrended. Finally, epochs with peak-to-peak differences >500 µM were excluded.

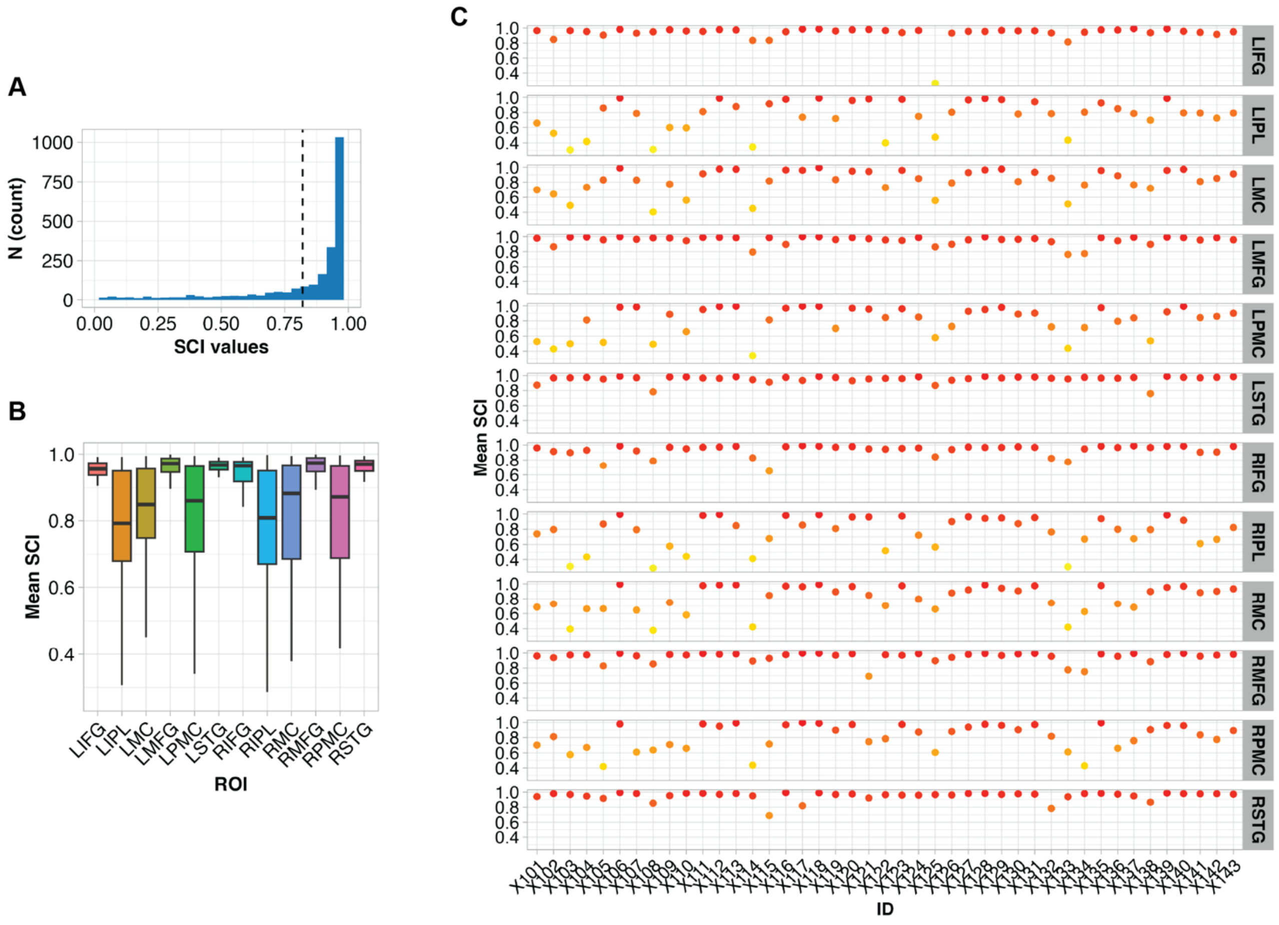

Figure 5. A) Histogram of scalp-coupling index (SCI) values for all channels. SCI was calculated for frequencies between 0.7-1.35 Hz (Pollonini et al., 2014). B) Boxplots per ROI, showing per-participant mean SCI values (i.e., distribution of values shown in panel C). The mean SCI per ROI ranges from 0.76-0.95, with median values ranging from 0.90-0.98. C) Mean SCI values per participant, per ROI.

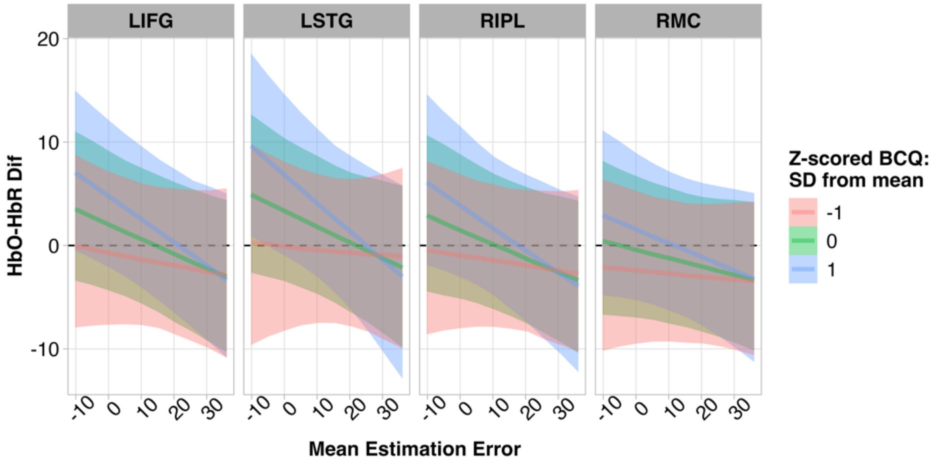

Figure 6. Predicted association between estimation error and cortical activation at different levels of body competence (BCQ), a self-report measure of embodiment. Shading shows 95% intervals of the posterior predictive distribution.
